# Effects of an 8-week resistance-to-CMJ training sequence on lower-body strength and countermovement-jump performance in resistance-trained men

**DOI:** 10.3389/fphys.2026.1982010

**Published:** 2026-10-29

**Authors:** Tingting Fu, Ziang Zhang, Jinchen Zhao, Faquan Li

**Affiliations:** 1 Faculty of Sports and Leisure, Guangdong Ocean University, Guangzhou, Guangdong, China; 2 China College of Basketball, Beijing Sport University, Beijing, China; 3 Department of Orthopedics, Wenzhou Central Hospital, Wenzhou, Zhejiang, China

**Keywords:** complex training, exercise sequencing, jump performance, maximal strength, plyometric training, resistance training

## Abstract

**Purpose:**

To compare the effects of 8-week resistance-to-plyometric (RT–PT) and plyometric-to-resistance (PT–RT) training sequences on lower-body strength, jumping, sprinting, and change-of-direction performance in resistance-trained men.

**Methods:**

In all, 24 resistance-trained men were randomly assigned to RT–PT or PT–RT training (n = 12 per group). Participants completed two supervised sessions per week for 8 weeks. Both groups performed three rounds of back squats (three repetitions at 85% 1RM) and maximal countermovement jumps (four repetitions), with 3 min of recovery between exercises and the exercise order reversed between groups. Training volume, relative intensity, and recovery intervals were matched. Vertical jump height (VJH), relative peak power (RPP), standing long jump (SLJ), back-squat one-repetition maximum (1RM), 20-m sprint time, and T-test time were assessed before and after training.

**Results:**

Significant improvements over time were observed for all outcomes (all p < 0.001). No significant group × time interactions were found for VJH, RPP, SLJ, 20-m sprint time, or T-test time (all p ≥ 0.501). Back-squat 1RM increased by 10.2 kg (95% CI: 7.5–13.0 kg) in RT–PT and 13.1 kg (95% CI: 12.1–14.1 kg) in PT–RT, with a significant group × time interaction in the primary analysis (p = 0.039, ηp^2^ = 0.180; Hedges’ g = 0.87, 95% CI: 0.01–1.73). However, this effect was not corroborated by the baseline-adjusted ANCOVA (p = 0.072).

**Conclusion:**

Both RT–PT and PT–RT training improved lower-body strength, jump, sprint, and change-of-direction performance. Exercise sequence had limited influence on long-term adaptations overall when training volume, relative intensity, and recovery intervals were matched. The sequence-related difference observed for maximal strength was not consistently supported across analyses and should therefore be interpreted cautiously.

## 1 Introduction

Explosive actions such as jumping, sprinting, accelerating, and performing changes of direction (COD) are important components of performance across many sports. These movements require athletes to generate high levels of force within a limited period, making maximal strength, rate of force development (RFD), and stretch-shortening cycle (SSC) function important determinants of physical performance (Suchomel et al., 2016). Heavy-load resistance training primarily enhances force-production capacity, whereas plyometric training emphasizes rapid eccentric-to-concentric transitions, elastic energy utilization, and force production at high movement velocities (Markovic and Mikulic, 2010). Because the adaptations induced by these training modalities are partly task-specific, combining resistance and plyometric exercises has become a common strategy for simultaneously developing strength, power, sprint, and COD performance (Cormie et al., 2010). Recent systematic reviews indicate that combined training provides a practical means of targeting multiple neuromuscular qualities within the same training program (Thapa et al., 2024a; Oliver et al., 2024).

Complex training involves performing a high-load resistance exercise before a biomechanically similar plyometric movement, such as completing a back squat before a countermovement jump (Cormier et al., 2022). The rationale for this resistance-to-plyometric sequence is partly based on post-activation performance enhancement, whereby a high-intensity conditioning activity may temporarily enhance subsequent explosive performance when potentiation-related effects exceed the accompanying fatigue (Blazevich and Babault, 2019; Seitz and Haff, 2016). When this high-force and high-velocity stimulus is repeated over several weeks, it may promote improvements in maximal strength and explosive performance. Meta-analytical evidence indicates that complex training can enhance vertical jump, sprint, COD, and maximal-strength performance (Thapa et al., 2024b; Cormier et al., 2020; Bauer et al., 2019). However, the effectiveness of this sequence depends on the balance between potentiation and fatigue. Heavy resistance exercise may produce residual neuromuscular fatigue that reduces the velocity, power output, and technical quality of the subsequent plyometric exercise, potentially limiting the intended explosive-training stimulus (Lloyd and Deutsch, 2008; Duthie et al., 2002).

Performing plyometric exercise before resistance exercise represents an alternative sequencing strategy. This plyometric-to-resistance sequence allows explosive movements to be completed in a relatively unfatigued state, which may help preserve movement velocity, power output, and technical quality. The subsequent resistance exercise can then provide the high-force stimulus required for maximal-strength development. Nevertheless, preceding plyometric exercise may also generate fatigue and reduce performance during the subsequent high-load resistance exercise. Therefore, each sequence may favor different components of the training session, and neither can be assumed to be universally superior (Ramirez-Campillo et al., 2020). Previous studies comparing resistance-to-plyometric sequences have generally reported improvements following both approaches, but the differences between them have been limited and inconsistent (Cormier et al., 2020; Kobal et al., 2017). More recently, an 8-week study in national-level basketball players found no consistent sequence-dependent differences in jump, sprint, or change-of-direction adaptations, although resistance-first training produced favorable changes in selected neuromuscular outcomes (Mikolajec et al., 2026). Despite these findings, limited research has compared the effects of resistance-to-plyometric training sequences while matching exercise selection, training volume, relative intensity, and recovery intervals.

Therefore, the purpose of this study was to compare the effects of an 8-week resistance-to-plyometric training sequence on lower-body strength and countermovement-jump performance in resistance-trained men. It was hypothesized that both training sequences would improve physical performance but that the magnitude of adaptation would differ between sequences.

## 2 Materials and methods

### 2.1 Participants

An *a priori* power analysis (α = 0.05, 1 − β = 0.80, f = 0.30) was performed using G*Power (version 3.1.9.7) for the group × time interaction in CMJ vertical jump height (VJH), indicating a required total sample size of 24 participants (actual power = 0.802). Twenty-four resistance-trained men (age: 22 ± 2.1 years, height: 179.8 ± 3.5 cm, body mass: 70.4 ± 5.5 kg) volunteered to participate in this study. All participants had at least 3 years of resistance-training experience (≥2 sessions/week) and were accustomed to performing a back-squat 1RM of at least 1.5 times body mass. Participants had been injury-free for at least 6 months before the study and did not complete any strenuous exercise in the 48 hours before each testing session. The study was approved by the *Wenzhou Central Hospital Review Board for Human Participants* (no. L2026-04-083) and complied with the principles of the *Declaration of Helsinki*. Written informed consent was obtained from all participants.

### 2.2 Study design

A randomized controlled design was employed to investigate the effects of two resistance–plyometric exercise sequences on physical performance. Following familiarization and pretesting, participants were randomly assigned to either a resistance-to-plyometric training group (RT–PT) or a plyometric-to-resistance training group (PT–RT). Participants completed intervention sessions two times per week over an 8-week period, with at least 72 h of recovery between sessions. Countermovement jump (CMJ), standing long jump (SLJ), Smith-machine back-squat one-repetition maximum (1RM), 20-m sprint performance, and the T-test were assessed before and after the intervention. Pre-intervention testing was completed at least 48 h before the first training session, whereas post-intervention testing was conducted at least 48 h after the final training session. Testing and training sessions were consistently performed at the same location and were supervised directly by the lead investigator. Environmental conditions were maintained at approximately 20 °C and 60% relative humidity, and all testing sessions were performed at the same time of day for each participant (± 1 h).

### 2.3 Resistance training program

The structure of the experimental visits is depicted in **Figure 1**. Participants completed 16 supervised training sessions over an 8-week period, with two sessions performed each week and at least 72 h between sessions. Each session began with a standardized warm-up consisting of 5 min of aerobic exercise, dynamic lower-limb stretching, two back-squat warm-up sets at 50% and 70% of the training load, and three submaximal CMJs. During each session, participants completed three rounds of back squats and CMJs. The RT–PT group performed three back-squat repetitions at 85% 1RM, followed by 3 min of passive recovery and four maximal CMJs. The PT–RT group completed the same exercises in the reverse order. A 4-min passive recovery period was provided between rounds. Back squats were performed on a Smith machine (Matrix Fitness, Cottage Grove, WI, USA) with a controlled eccentric phase of approximately 2 s and maximal intended concentric velocity. Participants descended until the thighs were approximately parallel to the floor and then returned to full hip and knee extension. All CMJs were performed with maximal effort while the hands remained on the hips. Training volume, relative intensity, and recovery intervals were matched between groups, with exercise order representing the only difference. Loads for weeks 1–4 were based on the preintervention 1RM and were recalculated after a mid-intervention 1RM test to maintain 85% 1RM during weeks 5–8. All sessions were supervised, and participants were required to complete at least 90% of the program.

**Figure 1:**
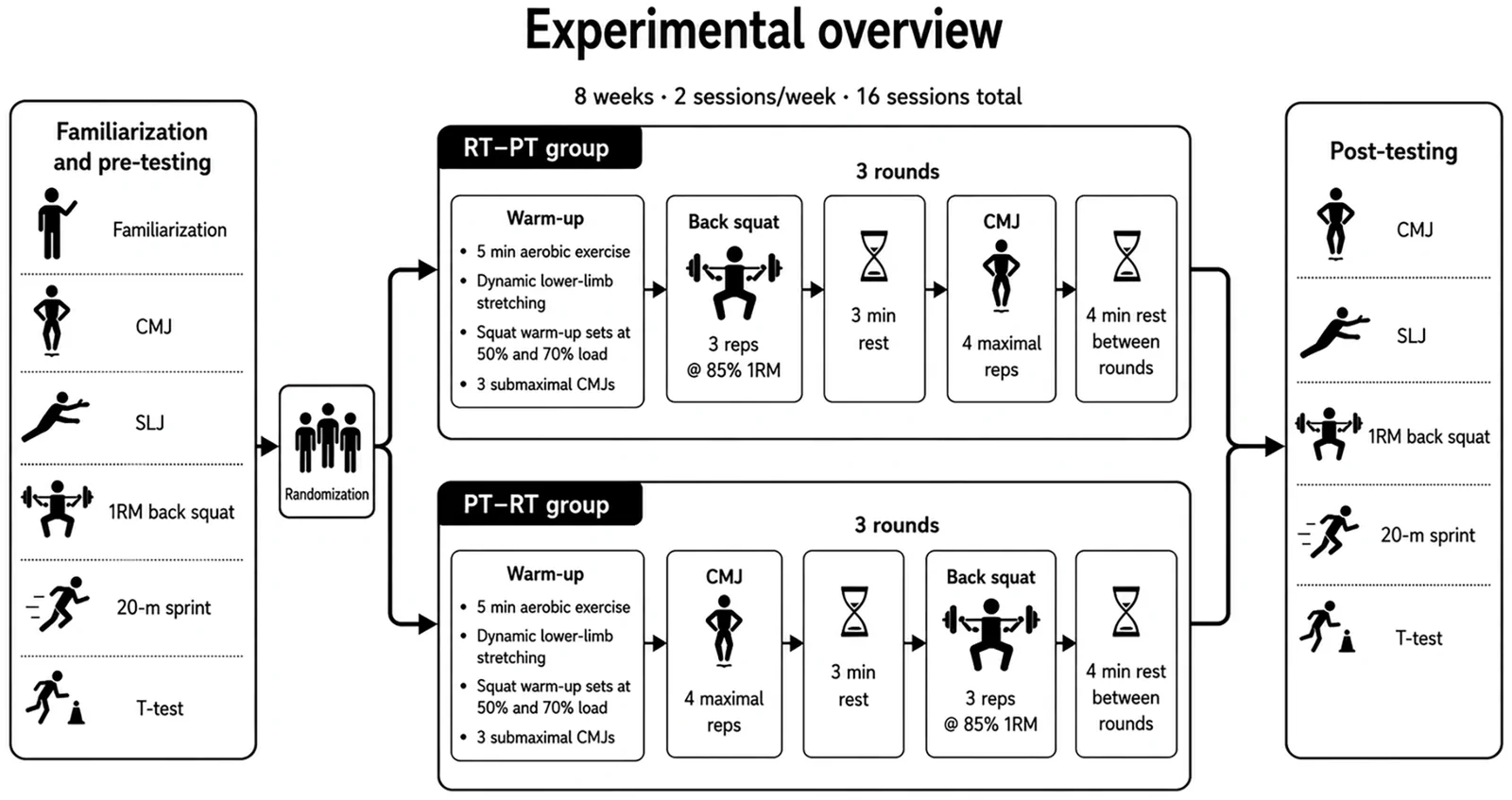
Experimental overview.

#### 2.3.1 One-repetition maximum testing

Back-squat one-repetition maximum (1RM) was assessed on a Smith machine in accordance with National Strength and Conditioning Association (NSCA) guidelines (Haff et al., 2016). Following 5 min of cycling (Power Max VIII; Konami Corporation, Tokyo, Japan) and dynamic stretching, participants completed 5 repetitions at approximately 50% of their estimated 1RM and 3 repetitions at approximately 70% of their estimated 1RM. Thereafter, single repetitions were performed with load increments of 5%–10% until the participant failed to complete the prescribed range of motion, defined as descending until the thighs were approximately parallel to the floor and returning to full hip and knee extension. Squat tempo was controlled using a metronome, with a 2-s eccentric and 1-s concentric phase. The highest successfully completed load was recorded as the 1RM. Testing was completed within five attempts, with 3 min of rest between attempts, under the supervision of certified strength and conditioning specialists (CSCS).

#### 2.3.2 Countermovement jumps

CMJs were performed on a force platform (model 9260AA; Kistler Corporation, Winterthur, Switzerland) sampling at 1000 Hz. Participants stood with their feet shoulder-width apart and hands on their hips, performed a rapid countermovement to approximately 90° of knee flexion, and then jumped vertically with maximal effort (Markovic et al., 2004). Three trials were completed with 60 s of rest between attempts, and the trial producing the greatest vertical jump height (VJH) was used for analysis. VJH was calculated from flight time between take-off and landing. Relative peak power (RPP) was derived from the force–time data and normalized to body mass. All variables were calculated using MARS software (version 4.0; Kistler Instruments Corp., Winterthur, Switzerland).

#### 2.3.3 20-m sprint testing

Participants completed three maximal 20-m sprints on an indoor running track, with 2 min of rest between trials. Sprint time was recorded using electronic timing gates (SmartSpeed; Fusion Sport, Coopers Plains, QLD, Australia) positioned at 0 and 20 m at approximately hip height (Cronin and Templeton, 2008). Participants started from a standing position with their preferred lead foot placed 1 m behind the first timing gate. The fastest 20-m sprint time was used for analysis.

#### 2.3.4 Change-of-direction ability

Change-of-direction (COD) ability was assessed using the T-test (Pauole et al., 2000), as illustrated in **Figure 2**. Four cones were arranged in a T-shaped configuration, with cone B positioned 10 m from the starting cone (A) and cones C and D positioned 5 m to either side of cone B. Participants sprinted from A to B, shuffled left to C, shuffled right to D, returned to B, and then backpedaled to A. Completion time was recorded using electronic timing gates (SmartSpeed; Fusion Sport, Coopers Plains, QLD, Australia) positioned at the start–finish line. Participants completed three trials with at least 2 min of rest between attempts, and the fastest time was used for analysis.

**Figure 2:**
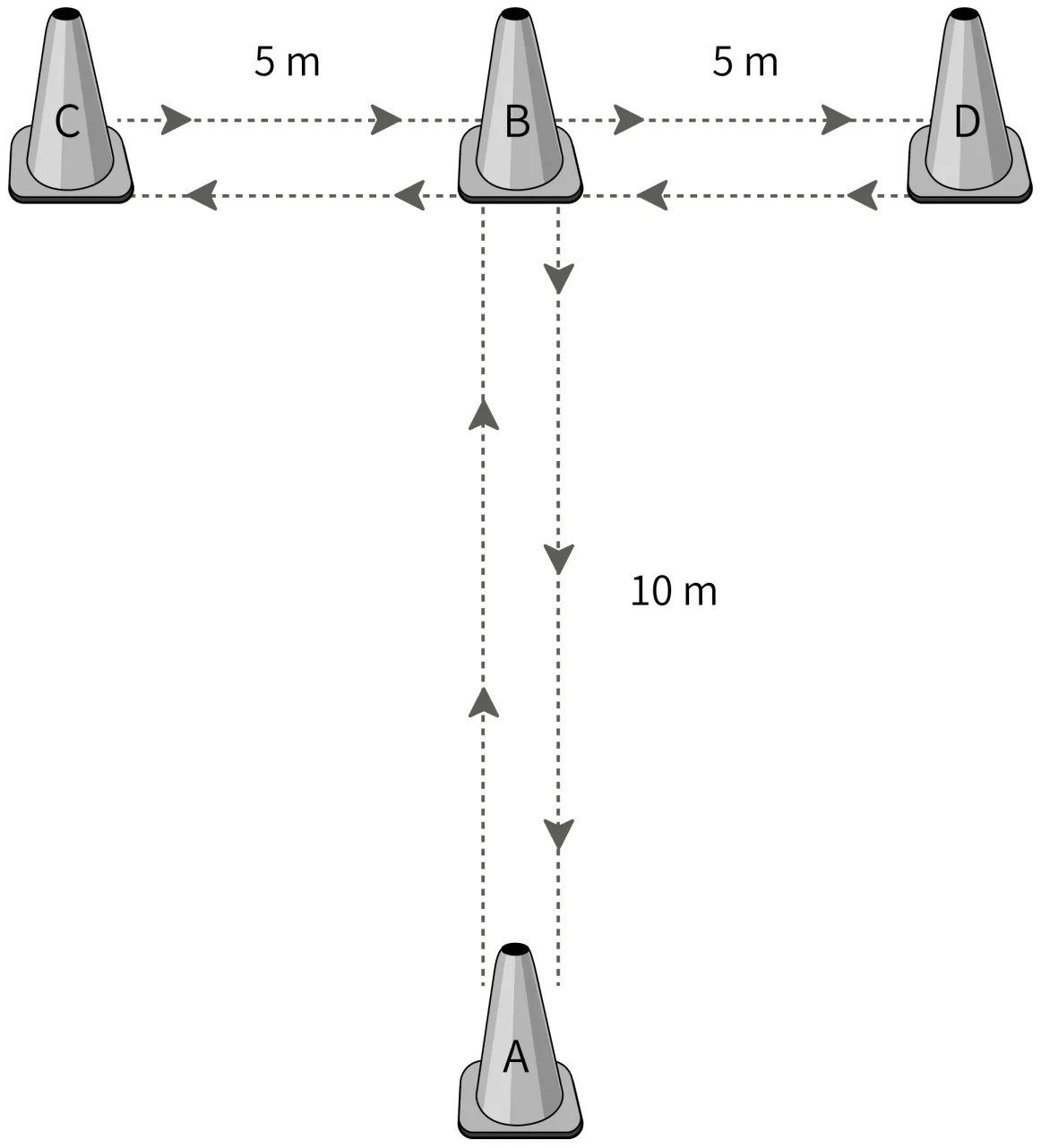
Schematic illustration of the T-test used to assess change-of-direction performance. Cone A represents the starting and finishing point, cone B is positioned 10 m from cone A, and cones C and D are positioned 5 m to either side of cone B. Participants followed the prescribed movement sequence A–B–C–D–B–A, incorporating forward sprinting, lateral shuffling, and backward running.

#### 2.3.5 Standing long jump

During the SLJ test, participants stood with their feet shoulder-width apart behind a marked starting line. On the command of “ready, set, go,” they executed a rapid countermovement, involving simultaneous knee flexion and arm swing, before explosively jumping forward as far as possible (Marin-Jimenez et al., 2024). The horizontal distance between the starting line and the heel of the rear foot was recorded using a tape measure to the nearest 1 cm. Participants completed three maximal trials, with a 60-s rest period between attempts. The best performance was recorded for further analysis.

### 2.4 Statistical analyses

Data are presented as mean ± SD. Normality of model residuals was assessed using the Shapiro–Wilk test, and homogeneity of variance was examined using Levene’s test. Separate 2 × 2 mixed-design analyses of variance were performed for VJH, RPP, SLJ, back-squat 1RM, 20-m sprint time, and T-test time, with group (RT–PT and PT–RT) as the between-subjects factor and time (pre- and post-intervention) as the within-subjects factor. Significant group × time interactions were followed by Bonferroni-adjusted pairwise comparisons. Partial eta-squared (ηp^2^) was calculated for the ANOVA effects. Hedges’ g was calculated for between-group differences in change with 95% confidence intervals. The mixed-design ANOVA was the prespecified primary analysis. Baseline-adjusted ANCOVAs were performed as complementary sensitivity analyses to account for baseline variation and assess the robustness of the primary ANOVA findings, with post-intervention values as the dependent variable, group as the fixed factor, and the corresponding pre-intervention values as covariates. Homogeneity of regression slopes was assessed using the baseline × group interaction. HC3-robust, Welch, and Wilcoxon analyses were additionally performed where relevant. Where a significant group × time interaction was observed, *post hoc* pairwise comparisons were performed using Bonferroni-adjusted p-values. A Bonferroni correction for the six group × time interaction tests was applied as a familywise sensitivity analysis. Statistical significance was set at p < 0.05. All analyses were performed using R version 4.5.2.

## 3 Results

Descriptive statistics, mean changes with 95% confidence intervals, interaction effects, and standardized between-group differences are summarized in **Table 1**. Individual pre- to postintervention changes are presented in **Figure 3**, whereas standardized between-group differences in change are illustrated in **Figure 4**. Baseline-adjusted ANCOVA and additional sensitivity analyses are reported in **Supplementary Table S1**.

**Table 1:** Pre- and post-intervention performance outcomes and between-group differences in change.

Variables	RT–PT (n = 12)			PT–RT (n = 12)			Group × time, F(1, 22), p, ηp^2^
	Pre	Post	Change (95% CI)	Pre	Post	Change (95% CI)	
VJH (cm)	46.8 ± 4.7	49.2 ± 5.9	2.4 (0.5 to 4.4)*	47.5 ± 5.9	50.2 ± 6.8	2.7 (1.8 to 3.6)*	0.08, 0.776, 0.004
RPP (W·kg^-^¹)	62.0 ± 2.2	64.8 ± 2.8	2.8 (1.6 to 4.0)*	62.4 ± 4.2	65.1 ± 4.7	2.7 (2.1 to 3.2)*	0.04, 0.849, 0.002
SLJ (m)	2.72 ± 0.05	2.79 ± 0.06	0.07 (0.05 to 0.10)*	2.68 ± 0.13	2.75 ± 0.12	0.07 (0.06 to 0.07)*	0.28, 0.604, 0.012
Back-squat 1RM (kg)	118.3 ± 10.8	128.5 ± 11.5	10.2 (7.5 to 13)*	125.2 ± 8.1	138.3 ± 9.0	13.1 (12.1 to 14.1)*	4.83, 0.039†, 0.180
20-m sprint time (s)	3.04 ± 0.07	2.97 ± 0.06	−0.06 (−0.08 to −0.05)*	3.04 ± 0.08	2.97 ± 0.07	−0.07 (−0.08 to −0.06)*	0.47, 0.501, 0.021
T-test time (s)	10.30 ± 0.27	10.16 ± 0.28	−0.14 (−0.21 to −0.06)*	10.39 ± 0.31	10.23 ± 0.30	−0.16 (−0.19 to −0.13)*	0.41, 0.531, 0.018

Values are presented as means ± SD. *Significant within-group change from preintervention to postintervention, p < 0.05. †Significant group × time interaction, p < 0.05. VJH, vertical jump height; RPP, relative peak power; SLJ, standing long jump; 1RM, one-repetition maximum; CI, confidence interval.

**Figure 3:**
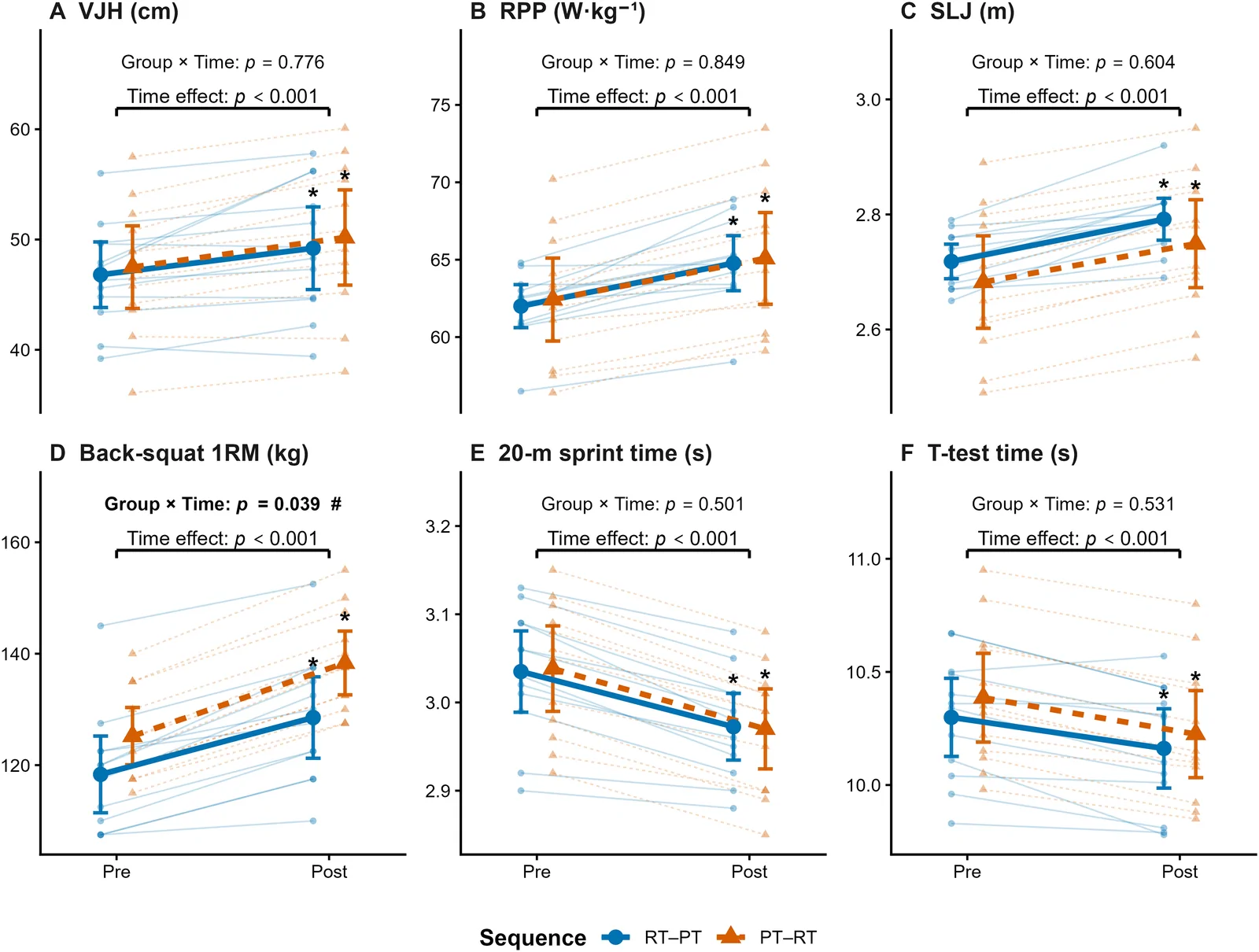
Individual pre- to post-intervention changes in physical performance following 8 weeks of resistance-to-plyometric (RT–PT) or plyometric-to-resistance (PT–RT) training. **(A)** Vertical jump height (VJH); **(B)** relative peak power (RPP); **(C)** standing long jump (SLJ); **(D)** back-squat one-repetition maximum (1RM); **(E)** 20-m sprint time; and **(F)** T-test time. Brackets indicate significant main effects of time (all p < 0.001), and the significant group × time interaction for back-squat 1RM is shown in bold. Lower values indicate better performance for the 20-m sprint and T-test.

**Figure 4:**
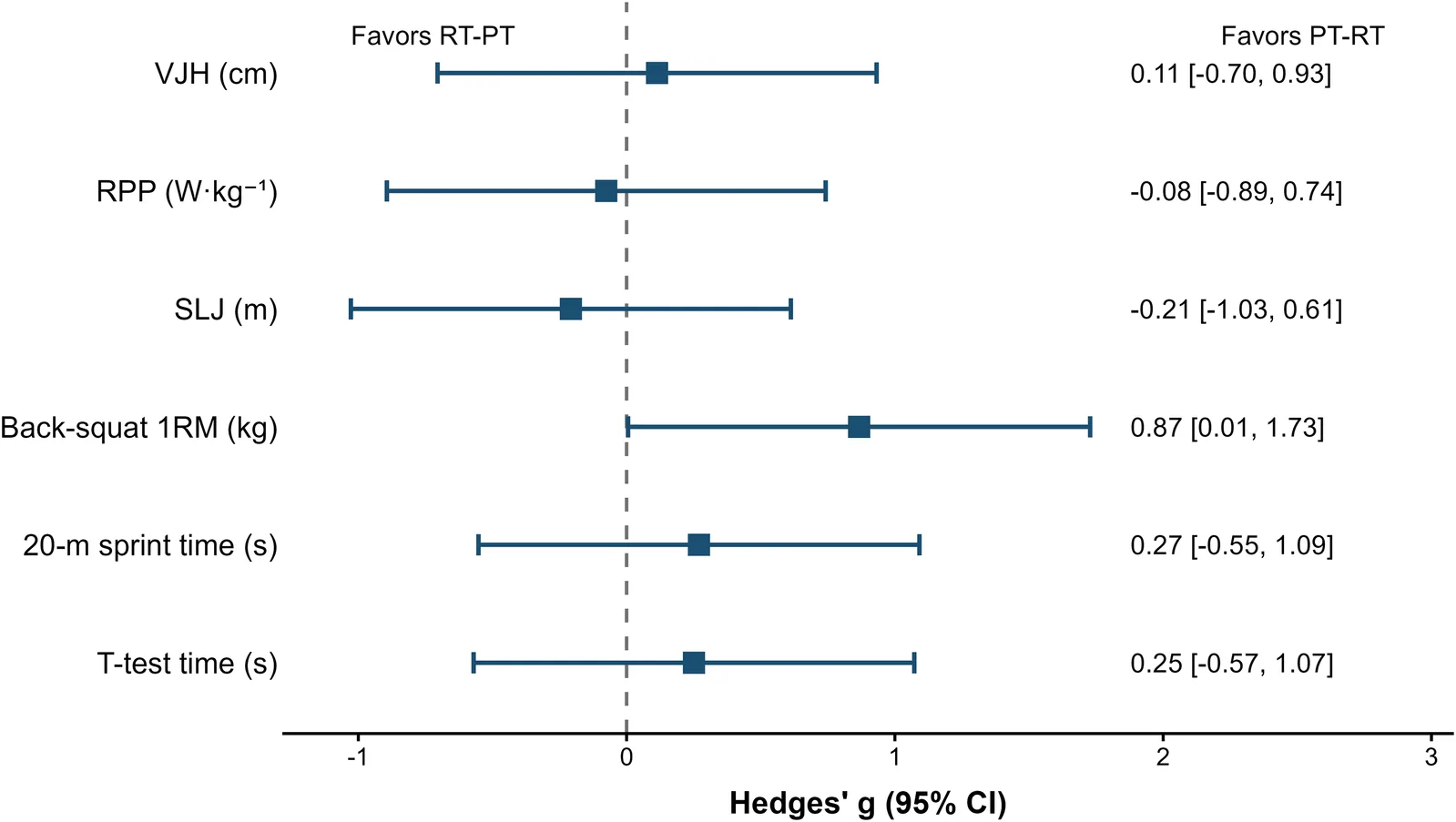
Standardized between-group differences in pre-to-post changes in physical performance. Squares represent Hedges’ g with 95% confidence intervals. Positive values favor the plyometric-to-resistance group (PT–RT), whereas negative values favor the resistance-to-plyometric group (RT–PT). For 20-m sprint and T-test outcomes, the direction of the effect sizes was reversed so that positive values consistently indicate greater improvement in PT–RT. The vertical dashed line indicates no between-group difference. Hedges’ g incorporates a small-sample correction; confidence intervals were calculated using the residual degrees of freedom for the independent comparison of change scores.

### 3.1 Countermovement-jump performance

Significant main effects of time were observed for VJH (F(1, 22) = 26.71, p < 0.001, ηp^2^ = 0.548) and RPP (F(1, 22) = 80.26, p < 0.001, ηp^2^ = 0.785). Mean VJH changes were 2.4 cm (95% CI: 0.5–4.4 cm) in RT–PT and 2.7 cm (95% CI: 1.8–3.6 cm) in PT–RT. Mean RPP changes were 2.8 W·kg^-^¹ (95% CI: 1.6–4.0 W·kg^-^¹) and 2.7 W·kg^-^¹ (95% CI: 2.1–3.2 W·kg^-^¹), respectively. No significant group × time interactions were observed for VJH (F(1, 22) = 0.08, p = 0.776, ηp^2^ = 0.004) or RPP (F(1, 22) = 0.04, p = 0.849, ηp^2^ = 0.002). Hedges’ g values for the between-group differences in change were 0.11 (95% CI: −0.70–0.93) for VJH and −0.08 (95% CI: −0.89–0.74) for RPP. The baseline-adjusted ANCOVA showed no significant group effects for VJH (p = 0.836) or RPP (p = 0.821).

### 3.2 Standing long-jump performance

A significant main effect of time was observed for SLJ (F(1, 22) = 122.42, p < 0.001, ηp^2^ = 0.848). Mean changes were 0.07 m (95% CI: 0.05–0.10 m) in RT–PT and 0.07 m (95% CI: 0.06–0.07 m) in PT–RT. The group × time interaction was not significant (F(1, 22) = 0.28, p = 0.604, ηp^2^ = 0.012). The between-group effect was Hedges’ g = −0.21 (95% CI: −1.03–0.61). Baseline-adjusted ANCOVA showed no significant group effect (p = 0.492).

### 3.3 Back-squat one-repetition maximum

A significant main effect of time was observed for back-squat 1RM (F(1, 22) = 309.38, p < 0.001, ηp^2^ = 0.934). Mean changes were 10.2 kg (95% CI: 7.5–13.0 kg) in RT–PT and 13.1 kg (95% CI: 12.1–14.1 kg) in PT–RT. A significant group × time interaction was observed (F(1, 22) = 4.83, p = 0.039, ηp^2^ = 0.180). The between-group effect was Hedges’ g = 0.87 (95% CI: 0.01–1.73). Welch and Wilcoxon sensitivity analyses yielded p = 0.045 and p = 0.046, respectively. Baseline-adjusted ANCOVA showed no significant group effect (p = 0.072). The Bonferroni-adjusted interaction value was p = 0.232.

### 3.4 20-m sprint performance

A significant main effect of time was observed for 20-m sprint time (F(1, 22) = 235.57, p < 0.001, ηp^2^ = 0.915). Mean changes were −0.06 s (95% CI: −0.08–−0.05 s) in RT–PT and −0.07 s (95% CI: −0.08–−0.06 s) in PT–RT. The group × time interaction was not significant (F(1, 22) = 0.47, p = 0.501, ηp^2^ = 0.021). The direction-standardized between-group effect was Hedges’ g = 0.27 (95% CI: −0.55–1.09). Baseline-adjusted ANCOVA showed no significant group effect (p = 0.478).

### 3.5 Change-of-direction performance

A significant main effect of time was observed for T-test time (F(1, 22) = 66.33, p < 0.001, ηp^2^ = 0.751). Mean changes were −0.14 s (95% CI: −0.21–−0.06 s) in RT–PT and −0.16 s (95% CI: −0.19–−0.13 s) in PT–RT. The group × time interaction was not significant (F(1, 22) = 0.41, p = 0.531, ηp^2^ = 0.018). The direction-standardized between-group effect was Hedges’ g = 0.25 (95% CI: −0.57–1.07). Baseline-adjusted ANCOVA showed no significant group effect (p = 0.619).

## 4 Discussion

The aim of this study was to compare the effects of 8 weeks of resistance-to-plyometric (RT–PT) and plyometric-to-resistance (PT–RT) training on lower-body strength and physical performance in resistance-trained men. Adaptations were driven primarily by the training intervention rather than exercise sequence, partially supporting our hypothesis. Both sequences improved VJH, RPP, SLJ, 20-m sprint time, and T-test performance, whereas sequence-dependent effects were limited to back-squat 1RM, for which the primary analysis showed greater improvement following PT–RT. However, this difference was not consistently supported by the baseline-adjusted ANCOVA or familywise sensitivity analysis. Overall, these findings indicate that when training volume, relative intensity, and recovery intervals are matched, exercise order has limited influence on long-term physical adaptations overall, although maximal-strength development may show some sequence-specific sensitivity.

### 4.1 Jump performance

Both RT–PT and PT–RT improved vertical and horizontal jump performance, with similar changes observed for VJH, RPP, and SLJ. The absence of significant group × time interactions and the trivial-to-small between-group effect sizes suggest that reversing the order of resistance and plyometric exercises did not meaningfully alter jump adaptations over the 8-week intervention. This finding is broadly consistent with previous evidence showing that different arrangements of resistance and plyometric exercises can produce comparable improvements in explosive performance when overall training volume and intensity are similar (Mikolajec et al., 2026; Kobal et al., 2017; Cormier et al., 2020). In particular, Kobal et al (Kobal et al., 2017). reported improvements in strength and jump performance following different combinations of strength, power, and plyometric training in young soccer players, whereas (Mikolajec et al., 2026) observed no consistent sequence-dependent differences in jump adaptations after 8 weeks of complex training in national-level basketball players. Similar observations have also been reported when plyometric training was performed before or after regular sport-specific training, suggesting that chronic jump adaptations may be relatively insensitive to exercise order when the total training stimulus is preserved (Ramirez-Campillo et al., 2020).

The improvements in both groups may reflect repeated exposure to complementary high-force and high-velocity stimuli. Heavy resistance training primarily targets maximal force-production capacity, whereas ballistic and plyometric exercises emphasize rapid force production and high movement velocity (Cormie et al., 2010; Markovic and Mikulic, 2010; Suchomel et al., 2016). Cormie et al (Cormie et al., 2010). demonstrated that heavy strength and ballistic power training induce partly task-specific adaptations, supporting the rationale for combining both stimuli when the objective is to improve multiple components of explosive performance. Meta-analytic evidence further indicates that combined or complex training can improve vertical-jump and power-related performance (Cormier et al., 2020; Bauer et al., 2019; Thapa et al., 2024a). In the present study, both groups completed identical back-squat and CMJ volumes, relative intensities, and recovery intervals, with exercise order representing the only experimental difference. The similar VJH and RPP adaptations therefore suggest that the cumulative exposure to high-force and high-velocity exercise may have been more influential than the order in which these stimuli were presented.

From an acute perspective, resistance-first sequencing is commonly justified by post-activation performance enhancement, whereby a high-intensity conditioning activity may transiently enhance subsequent explosive performance if potentiation-related effects outweigh fatigue (Blazevich and Babault, 2019; Seitz and Haff, 2016). However, heavy resistance exercise can also produce residual fatigue and reduce subsequent jump or power performance (Lloyd and Deutsch, 2008; Duthie et al., 2002). Conversely, performing plyometric exercise first may preserve explosive-movement quality because the jumps are completed before exposure to heavy resistance exercise. Previous acute work has shown that the order of loaded squats and explosive exercises can modify the immediate performance response (Duthie et al., 2002). Nevertheless, the present findings indicate that these acute sequence-dependent effects did not translate into clearly different long-term adaptations in VJH or RPP.

The similar improvement in SLJ is also noteworthy because the training intervention employed CMJs rather than SLJs. This suggests that the adaptations induced by repeated heavy squatting and maximal jumping transferred to an untrained jumping task. Improvements in lower-body force-production capacity and explosive performance may contribute to both CMJ and SLJ performance (Markovic and Mikulic, 2010). However, the lack of a between-group difference indicates that this transfer was not meaningfully influenced by exercise sequence.

### 4.2 Maximal strength

Both training sequences produced substantial improvements in back-squat 1RM, indicating that repeated exposure to high-load squatting at 85% 1RM was effective for developing maximal strength. Unlike the other outcomes, however, the primary mixed-design analysis revealed a significant group × time interaction for 1RM, with a larger improvement following PT–RT. The between-group effect size was also relatively large, suggesting that exercise order may have influenced maximal-strength adaptation under the present training conditions.

One possible explanation is that performing a small number of maximal CMJs before the resistance exercise did not produce sufficient fatigue to impair subsequent high-load squatting. Previous acute studies have shown that the performance response to different strength–power sequences depends on the interaction between potentiation and fatigue (Duthie et al., 2002; Lloyd and Deutsch, 2008). Duthie et al. (2002) demonstrated that heavy and explosive exercises can interact differently depending on their sequencing, while Lloyd and Deutsch (2008) reported that changing the order of loaded squat and countermovement-jump exercises altered acute performance responses during complex training. Similarly, the magnitude of post-activation performance enhancement has been shown to depend on factors such as training status, conditioning activity intensity, and recovery duration (Blazevich and Babault, 2019; Seitz and Haff, 2016). It is therefore possible that the four CMJs performed before the squat in PT–RT provided an acute preparatory stimulus without substantially impairing subsequent resistance performance, although this possibility could not be directly verified in the present study.

Another possible explanation is related to exercise quality. Performing plyometric exercise at the beginning of the training sequence may allow athletes to complete the explosive component with minimal prior fatigue, while the subsequent 3-min recovery period may provide sufficient time before the heavy squat. Conversely, in RT–PT, residual fatigue from the high-load squats may primarily affect the subsequent plyometric exercise rather than the strength stimulus itself. Acute studies have shown that heavy resistance activity can reduce subsequent velocity or power output when recovery is insufficient (Lloyd and Deutsch, 2008). Over repeated training sessions, small differences in exercise quality could theoretically accumulate and influence adaptation. However, this explanation remains speculative because barbell velocity, repetition velocity loss, CMJ output during training, and neuromuscular activity were not monitored in the present study.

The 1RM finding should also be interpreted in the context of the sensitivity analyses. Although the primary mixed-design ANOVA and both Welch and Wilcoxon change-score analyses supported a sequence-dependent difference, the baseline-adjusted ANCOVA did not reach statistical significance, and the interaction was no longer significant after familywise Bonferroni adjustment. Thus, the present data provide some indication that PT–RT may favor maximal-strength adaptation, but they do not provide definitive evidence of its superiority. This is particularly relevant because previous longer-term studies have generally reported limited or inconsistent effects of resistance–plyometric exercise order on physical-performance adaptations (Cormier et al., 2020; Ramirez-Campillo et al., 2020; Mikolajec et al., 2026). Future studies with larger samples and direct monitoring of training velocity and repetition quality are needed to determine whether the strength difference observed here represents a reproducible sequencing effect.

### 4.3 Sprint and change-of-direction performance

Both training sequences improved 20-m sprint and T-test performance, whereas no significant group × time interactions were observed for either outcome. These findings are consistent with previous evidence showing that combined resistance and plyometric training can improve sprint and COD performance (Thapa et al., 2024a, 2024b; Cormier et al., 2020; Bauer et al., 2019), while the specific order of the resistance and explosive components does not consistently produce different long-term adaptations (Cormier et al., 2020; Kobal et al., 2017; Mikolajec et al., 2026). Mikolajec et al. (2026), for example, reported improvements in sprinting and change-of-direction performance following different complex-training arrangements, but found no consistent advantage for one exercise sequence.

The observed improvements may partly reflect transfer from increases in lower-body force-production and explosive capabilities. Sprint acceleration requires the rapid application of substantial force, while COD performance additionally depends on braking, reacceleration, and the ability to rapidly redirect force (Suchomel et al., 2016). Resistance training can improve force-production capacity, whereas plyometric training may enhance rapid force application and stretch-shortening cycle function (Suchomel et al., 2016; Markovic and Mikulic, 2010). Consequently, repeated exposure to both high-force squatting and maximal jumping may have contributed to the observed improvements even though sprinting and COD drills were not directly included in the intervention.

However, the transfer from squat and CMJ training to sprinting and COD is less task-specific than the transfer to jumping performance. The intervention did not include sprint-specific acceleration work, lateral plyometrics, deceleration drills, or planned change-of-direction exercises; instead, both groups performed the same back-squat and CMJ training throughout the 8-week period. This may explain why the improvements in sprint and COD were similar between groups. Any subtle sequence-dependent differences in the acute training stimulus may have been insufficient to produce distinguishable adaptations in these more complex locomotive tasks.

The lack of a sequencing effect also agrees with the broader literature suggesting that total training content may be more important than exercise order for chronic sprint and COD adaptations. Systematic reviews of complex and combined training have generally reported favorable effects on sprint and COD performance but considerable variability between studies according to exercise selection, loading, recovery, and training status (Thapa et al., 2024b; Cormier et al., 2020; Bauer et al., 2019). Therefore, when the volume and relative intensity of resistance and plyometric work are closely matched, the order in which they are performed may represent only one of several factors influencing transfer to sprint and COD performance.

## 5 Limitations and additional considerations

Several limitations should be acknowledged. First, the sample consisted exclusively of resistance-trained young men, limiting the generalizability of the findings to women, youth athletes, elite athletes, and individuals with different training backgrounds. Second, the intervention lasted only 8 weeks and employed a single training configuration consisting of Smith-machine back squats at 85% 1RM paired with maximal CMJs; therefore, the findings may not apply to longer training periods, different loading intensities, free-weight exercises, or other plyometric movements. Additionally, the CMJ may not meet all definitions of a plyometric exercise because it lacks a preceding flight phase and typically involves a relatively long ground-contact time. Third, training-session performance was not directly monitored using measures such as barbell velocity, within-set velocity loss, CMJ output, or neuromuscular activity. Consequently, we cannot determine whether exercise sequence altered acute fatigue, movement-execution quality, or neuromuscular activation during the paired exercises. Therefore, the proposed mechanisms underlying the sequence-related responses remain speculative. Fourth, the relatively small sample size may have limited the ability to detect modest between-group differences. Finally, although the primary analysis indicated a sequence-dependent effect for back-squat 1RM, this finding was not consistently supported by the baseline-adjusted ANCOVA or the familywise-adjusted sensitivity analysis and should therefore be interpreted cautiously.

### 5.1 Practical applications

RT–PT and PT–RT can both be used as practical training strategies to improve lower-body strength, jumping, sprinting, and change-of-direction performance in resistance-trained men. Based on our findings, coaches may select the exercise sequence according to the primary emphasis of the session, athlete preference, or practical constraints when training volume, relative intensity, and recovery intervals are matched. RT–PT may be preferred when the aim is to perform explosive movements after a high-force stimulus, whereas PT–RT may be used when preserving plyometric quality early in the session is prioritized. Although the primary analysis indicated a greater improvement in back-squat 1RM following PT–RT, this effect was not consistently supported by the complementary analyses. Accordingly, the apparent sequence effect on maximal strength should be regarded as tentative rather than as evidence of a clear advantage of PT–RT. Overall, exercise sequence appears to be a secondary programming consideration compared with the total exposure to high-force and high-velocity stimuli.

## 6 Conclusion

Eight weeks of resistance–plyometric training improved lower-body strength, CMJ and SLJ performance, sprint performance, and change-of-direction ability. Adaptations were generally similar between sequences, although a sequence-related difference in back-squat 1RM emerged in the primary analysis and should be interpreted cautiously given its limited robustness across complementary analyses. Overall, exercise order appears to have limited influence on long-term adaptations when training volume, relative intensity, and recovery intervals are matched.

## Data Availability

The raw data supporting the conclusions of this article will be made available by the authors, without undue reservation.
